# Effect of Physiological Fluids Contamination on Selected Mechanical Properties of Acrylate Bone Cement

**DOI:** 10.3390/ma12233963

**Published:** 2019-11-29

**Authors:** Robert Karpiński, Jakub Szabelski, Jacek Maksymiuk

**Affiliations:** 1 Department of Machine Design and Mechatronics, Faculty of Mechanical Engineering, Lublin University of Technology, Nadbystrzycka 36, 20-618 Lublin, Poland; 2 Section of Biomedical Engineering, Department of Computerization and Production Robotization, Faculty of Mechanical Engineering, Lublin University of Technology, Nadbystrzycka 36, 20-618 Lublin, Poland; 3 Orthopaedic Department, Łęczna Hospital, Krasnystawska 52, 21-010 Łęczna, Poland

**Keywords:** bone cement, contamination, human blood, saline solution, biodegradation, mechanical properties, compressive strength, hardness

## Abstract

This study analyses the degradation rate of selected mechanical properties of bone cement contaminated with human blood and saline solution. During the polymerisation stage, the PMMA cement specimens were supplemented with the selected physiological fluids in a range of concentrations from 0% to 10%. The samples were then subjected to the standardised compression tests, as per ISO 5833: 2002, and hardness tests. The obtained results were analysed statistically to display the difference in the degradation of the material relative to the degree of contamination. Subsequently, numerical modelling was employed to determine the mathematical relationship between the degree of contamination and the material strength degradation rate. The introduction of various concentrations of contaminants into the cement mass resulted in a statistically significant change in their compressive strength. It was shown that the addition of more than 4% of saline and more than 6% of blood (by weight) causes that the specimens exhibit lower strength than the minimum critical value of 70 MPa, specified in the abovementioned International Standard. It was further revealed that the cement hardness characteristics degraded accordingly. The mathematical models showed a very good fit with the results from the experiments: The coefficient of determination R^2^ was 0.987 in the case of the linear hardness model for blood and 0.983 for salt solution; secondly, the values of R^2^ for the third-degree polynomial model of compressive strength were 0.88 for blood and 0.92 for salt. From the results, it can be seen that there is a quantitative/qualitative relationship between the contamination rate and the drop in the tested mechanical characteristics. Therefore, great effort must be taken to minimise the contact of the bone cement with physiological fluids, which naturally occur in the operative field, particularly when the material cures, in order to prevent the cement material strength declining below the minimum threshold specified in the ISO standard.

## 1. Introduction

The term “bone cement” refers to artificial bone materials based on polymethyl methacrylate (PMMA) or other acrylate-based polymers [1,2,3]. The scope of medical applications of cement materials primarily involves fixing endoprostheses in total knee arthroplasty (TKA) and total hip arthroplasty (THA). This medical substrate is, moreover, applied in reconstructive procedures, particularly in the presence of bone defects that require grafting, such as in the case of vertebroplasty and kyphoplasty [4,5,6,7,8,9,10]. Note that cement is not an adhesive material, and it does not form a permanent bond with the implant or the bone. Therefore, it is the interlocking, i.e., the formation of a bone-cement composite by introducing cement into spaces in the bone prior to its polymerisation that determines its essential applicability in the given scope [11]. In implants, the cement supports the prosthesis and ensures uniform distribution of stress accompanying the patient’s movement. Therefore, this study set out to determine whether contamination of bone cement may facilitate its accelerated crumbling, as a result of which it would cease to perform its primary function, which is to provide support to the prosthesis, and could eventually lead to the loosening of the implant.

Despite their ongoing dynamic evolution of bone surgery biomaterials, cements continue to fall behind the expected elasticity, fatigue and fracture strength requirements. The properties of the polymethyl methacrylate (PMMA) bone cement earmark it as an effective arthroplasty and bone-grafting solution. However, due to its disadvantages, such as non-biodegradability and osteogenesis, the scope of its clinical applications is limited [12,13,14,15,16]. Bone cement is regarded to be the weakest link in the bone-cement-implant system; therefore, determining the mechanical characteristics of these materials may prove decisive to the success or failure of an orthopaedic procedure. The mechanical properties of the material under investigation are decreased by such factors as, inter alia, elevated temperature, humidity, ageing, the inclusion of contrast agents and antibiotics. The basic strength properties, crack resistance or fatigue and creep strength are also strongly associated with the implantation process and factors pertaining to the human body environment, such as the admixture of blood and physiological fluids or the presence of bone tissue residues in the cement structure [17,18,19,20,21]. The cement preparation technique [22] and the conditions in the operative field have been also found to be of considerable importance. Despite their significance, several factors determine that the appropriate conditions are not ensured, thus facilitating bone contamination; these include substandard work organisation, hospital conditions, financial issues or the orthopaedist’s habits. Other material, e.g., the patient’s blood, fragments of the adipose tissue, synovial fluid, not to mention the 0.9% NaCl solution, which is routinely used to rinse the joint prior to implantation, may further compromise the properties of the cement. Therefore, the proper preparation of bones and the surgical field for implant placement is of the highest priority. During implantation, the use of a tourniquet band [23], pulse lavage and accurate bone drying should be observed in all cases. Investigating the effect of bone cement contamination on its properties further highlights the need to follow these procedures.

Implant fixation with bone cement is an established method in modern knee arthroplasty; however, obese, young or physically active patients might be exposed to a greater risk of loosening compared with cementless implants [24]. Changes in the mechanical properties of cement, and the resulting implant loosening, frequently necessitate revision surgeries and replacement of the endoprosthesis [12,25,26,27,28]. In 2016, more than 860,000 primary knee replacement procedures were performed in the USA alone. Their revision rate amounts to approximately 4%. Based on the available data, the most common cause of revision surgeries is aseptic loosening, being responsible for 20% to 40% of procedures [29]. Revision procedures are the source of numerous disadvantages, including a longer operating time, greater blood loss, higher risk of complications, prolonged hospitalisation, multiple operations, increased hospital overhead costs and, above all, the discomfort of patients resulting from the need to return to outpatient departments, often for paid visits [30].

To establish a set of critical features of materials used in orthopaedic surgery, theoretical and experimental methods must be employed. Conducting experiments on humans is both a difficult and time-consuming process and should be limited for ethical reasons, which is why basic research works that involve the use of physical and numerical models could be used to provide an accurate representation of the actual operating conditions of the investigated objects [22,31,32,33]. Therefore, with the aim of broadening the perspective on the problem, the properties of bone cements and their changes should not be necessarily tested in surgery.

**Figure 1:**
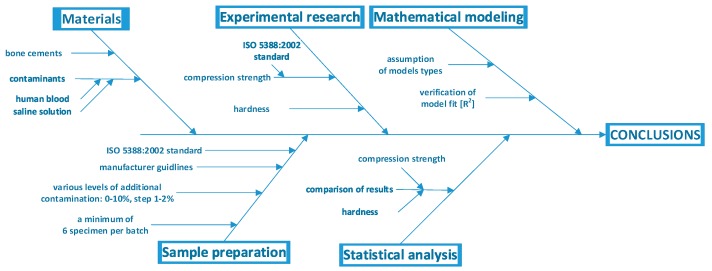
Flowchart describing the study methodology.

Bearing in mind the abovementioned, this study attempts to analyse the behaviour of the bone cement material under contamination with selected physiological fluids (human blood and saline solution). Specifically, the reported study focused on the degradation process of the most essential characteristics of cement strength: Its compression strength and hardness (Figure 1).

Far too little attention has been paid to the effect of contamination on the cement strength properties, in particular in the presence of human blood [34]. The focus of bone-cement studies is on admixtures of auxiliary agents, supplied to improve the material’s properties, including strength [22,26,33,35,36,37,38].

## 2. Materials and Methods

### 2.1. Substrate and Sample Preparation

The material under analysis in the reported study was DePuy CMW 3 GENTAMYCIN bone cement powder (Raynham, MA, USA), composed of powder (Gentamicin Sulphate 4.22%, Polymethyl Methacrylate 83.88%, Benzoyl Peroxide 1.9%, Barium Sulphate 10%) and liquid (Methyl Methacrylate 97.5%, *N*,*N*-Dimethyl-p-toluidine < 2.5%, Hydroquinone 75 ppm). The ϕ6 mm × 12 mm cylindrical test specimens were moulded in accordance with the International Standard 5833: 2002 (Implants for surgery—acrylic resin cements). When a height discrepancy from the standard was revealed, the specimens were subjected to subtractive machining: The samples were made with allowance and then ground to the target dimension of 12 mm. Moreover, this procedure ensured that the cylindrical sample bases were coplanar at both ends and perpendicular to the specimen’s axis of symmetry. The samples also underwent quality control—those exhibiting faults in the substrate, e.g., in the cylinder volume and other visible structural defects, were replaced with the correct ones. Prior to joining, both substrates, i.e., the powder and fluid, were cooled to 16 °C to extend the polymerisation time as a tactic to improve the precision of filling the mould. The tests were carried out for different cases of quantitatively variable degree of cement mass contamination, in the range of 0% to 10% by weight. The specimens were tested at 23 °C. Each batch consisted of at least 6 specimens, which is more than the minimum number specified in the referenced standard. Increasing the number of samples could potentially further improve the accuracy of the obtained results. In all batches, the specimens were produced from identical cement packs.

The source of contamination in the cement composition was the physiological fluid, the presence of which is unavoidable during the implantation of bone cement prostheses. The physiological saline used in the study was a typical, commercial, 0.9% isotonic saline solution, whose applications include intravenous fluid replenishment in the blood vessel system, washing wounds or moistening tissues and cleaning the operative field during surgical procedures. Due to the planned testing of human blood specimens, the project (i.e., the research plan and the materials) underwent the assessment of the Scientific Research Ethics Committee of the Lublin University of Technology, as required by the Order No. R-51/2015 of the Rector of Lublin University of Technology of 21 October 2015. Having obtained a positive opinion and approval (resulting from Resolution No. 5/2016 of 19 December 2016) the research could proceed. Prior to blood donation, the donor was informed in detail on the course of the donation procedure, as well as possible contraindications and consequences, upon which the informed consent was given. The A+ blood was collected from a single puncture into the vein of the upper limb in the area of the elbow flexion. The skin at the injection site was primarily disinfected with an ethyl alcohol solution. The blood samples were collected without the presence of anticoagulants, to realistically recreate the conditions during surgery. All persons who had contact with potentially infectious biological material used personal protective equipment. The procedures concerning the use of potentially infectious material were supervised by a doctor of medicine. The laboratory devices in contact with potentially infectious material were disinfected with a multi-purpose washing/disinfecting agent. All disposable devices were disposed of as medical waste. The blood was obtained in a sterile manner by a qualified and authorized physician and immediately used for sample preparation. The blood was not modified in any way or stored over the period exceeding the coagulation time.

It was resolved that the contaminants should be introduced into the cement mass at the material preparation stage, which was dictated by two factors: First, soaking the samples in blood or saline would only contaminate the periphery of the specimen and, secondly, an additional factor—time—would appear in the tests. The longer the soaking, the greater and the deeper the absorption (as shown in our former study: [22]). It is for these reasons that full-contamination testing was undertaken. For this purpose, a specified weight amount of contaminant was dosed into a constant amount of uncured cement mass and mixed. For clarity, in further analysis, the content of contamination in the cement is given in relative units—% w/w. Note that it is typically enough for the cement mass located in the intramedullary canal and around the prosthesis (treated as a whole) to become degraded by blood at the interface with the bone alone to cause aseptic loosening of the entire “assembly”. The procedure has allowed us to investigate the extreme scenario, in which the cement strength is compromised under the influence of contamination.

### 2.2. Compressive Strength and Hardness Tests

The effect of the physiological fluid contamination on the selected strength properties of the tested bone cement was evaluated in physical tests, which consisted of compressive strength and surface microhardness tests. The compression strength tests were performed with the use of test stand based on the MTS Bionix–Servohydraulic Test System for biomedical material testing applications (Eden Prairie, MN, USA). The course of the experiment was programmed and executed by MTS TestWorks software (Eden Prairie, MN, USA). The compression speed of the samples was specified as per the International Standard ISO 5833:2002 (Implants for surgery–acrylic resin cements) at 20 mm/min. During the test, the minimum breaking loads were recorded and converted to stresses for further analysis (the force divided by the original cross-sectional area of the cylinder).

**Figure 2:**
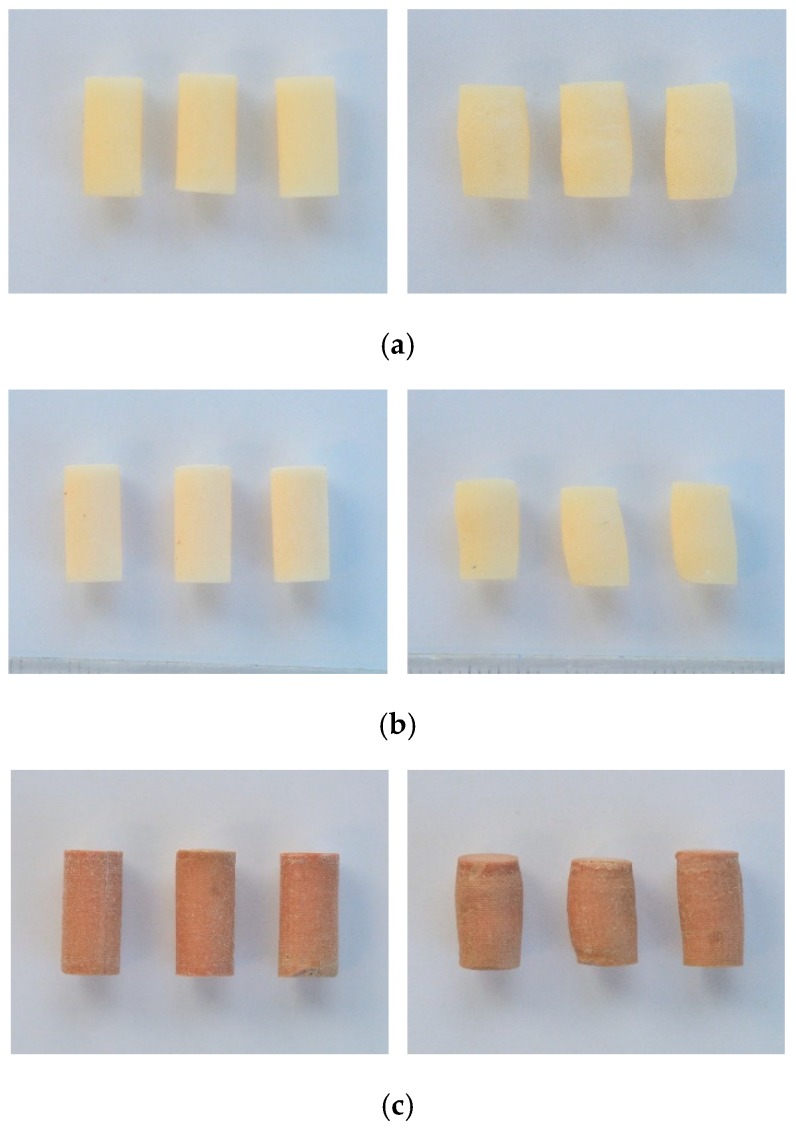
Sample pre- (**left**) and post-test (**right**) specimens: (**a**) non-contaminated, (**b**) contaminated with saline solution (~8% w/w), and (**c**) contaminated with human blood (~8% w/w).

With respect to the cement specimens’ hardness, it was subjected to Shore static hardness measurement method for rigid plastics, based on the Standard PN EN ISO 868:2005 (Plastics and ebonite—determination of indentation hardness by means of a durometer (Shore hardness)). The tests were carried out using an AFFRI durometer for polymeric materials connected with a manual bench support ART 13 with a resolution of 0.1° Shore, with an electronic processor and interchangeable probes (Induno Olona, VA, Italy). The testing was preceded by the visual inspection of the specimens to verify whether the samples were dented or deformed at specified measuring points, followed by cleaning with an alcohol-soaked cloth. During the measurements, due care was taken to ensure that the tested specimen is perpendicular to the probe. Based on preliminary tests, the Shore D scale was determined to be the most appropriate for the measurements. The tests were carried out with a 30° A-shaped cone, the applied force was equal to 44.5 N and each measurement was performed in 8 repetitions. The distance between the measuring points on the specimens was 10 mm of each other and less than 9 mm from the specimen edge. The accuracy of the performed measurements was ascertained by following the standardised test procedure: After reaching the state of equilibrium between the indenter pressure and the reaction of the tested material, a 15 s measurement was taken. The sample cement specimens before and after the compressive strength test are shown in Figure 2. At subsequent cement mass contamination levels, the colour change of the specimens with the addition of blood was noted. The specimens contaminated with the saline solution did not show such a pronounced discolouring [39].

### 2.3. Statistical Analysis and Mathematical Modelling

The results obtained from the tests of selected mechanical properties of the cement specimens were subjected to statistical analysis in order to provide the confirmation and quantification of the effect of selected physiological fluids on the selected mechanical properties. The statistical works additionally included the preparation of a mathematical model showing the relationship between the analysed parameters and the obtained results. The following packages were used to this end: TIBCO Software Inc. (Palo Alto, CA, USA) (2017); Statistica (data analysis software system), version 13, http://statistica.io (Palo Alto, CA, USA); and Microsoft Excel 2013 (Redmond, WA, USA).

To determine whether there exists a statistical significance between the sets of data obtained from the compression strength and the material hardness tests (including the scatter of results within each series) depending on the degree of contamination, a detailed statistical analysis was carried out, with a standard level of statistical significance α = 0.05. The normality of distribution of the obtained results was analysed by means of three tests: Kolmogorov–Smirnov, Lilliefors and Shapiro–Wilk. Subsequently, the homogeneity of variances was tested with the F (Fisher), Levene, Brown and Forsyth tests; relative to their results, further analyses would include tests of equality of means—the *t*-Student test or the Cochran–Cox test (i.e., Student test with a separate-variance adjustment).

In order to fully understand the relation between the analysed mechanical properties of the material and the effect of contamination in the cement structure, the mathematical modelling was performed with Statistica 13.1 software, based on the compressive strength and hardness test results. The potential distribution of the obtained measurement values was estimated, on the basis of which the linear or polynomial model type was preselected. The quality of the obtained models was verified by means of the coefficient of determination R^2^ for an individual model. Better-fitting models, showing high R^2^, were employed when needed. Such modelling allows projecting, to a certain extent, the behaviour of the material in long-term operation.

## 3. Results

### 3.1. Compression Strength

**Figure 3:**
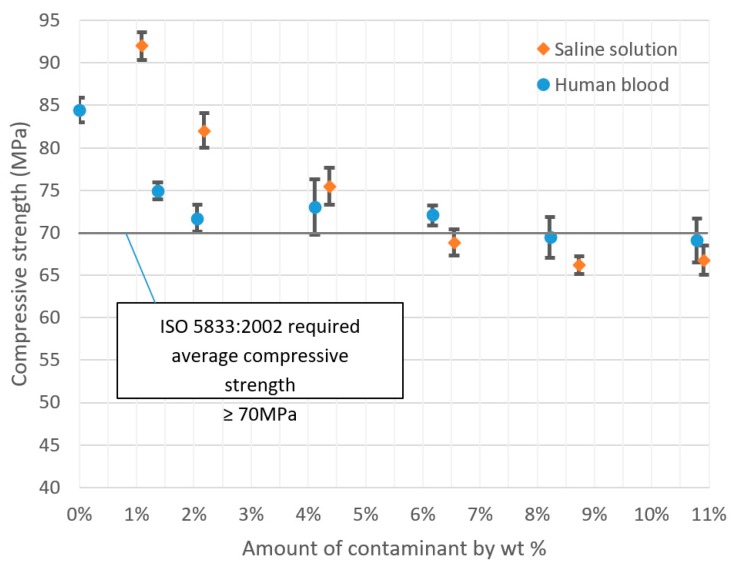
Decrease in compressive strength of bone cements relative to the degree of contamination with physiological fluids.

**Figure 4:**
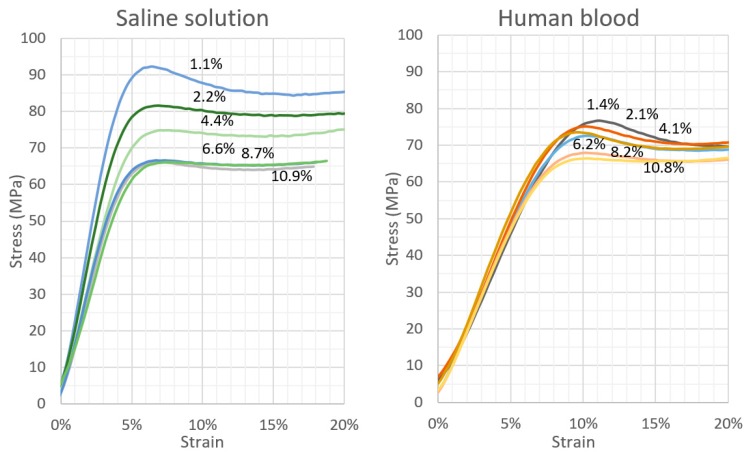
Stress–strain curves for sample specimens of every batch.

The results of the compressive strength tests of the cement samples with varying degrees of physiological fluid contamination are presented in Table 1 and Table 2. Note that all the measurements exhibit an exceptionally low scatter of results, and the coefficient of variation is below 5% in each scenario. This indicates a high homogeneity of the tested properties within a test series. Figure 3 presents, in the graphic form, the course of changes in the tested parameters. The decrease in the average compressive strength of cement samples clearly increases with the rising amount of contamination in the mix; however, it is in the case of the physiological saline solution that it is noticeably greater than for the human blood. The presence/absence of statistically significant differences between successive series of samples was verified with the application of detailed statistical analyses of the results, which are presented in the further sections of this report. Figure 4 presents sample stress–strain curves for selected specimens of every batch.

### 3.2. Hardness

**Figure 5:**
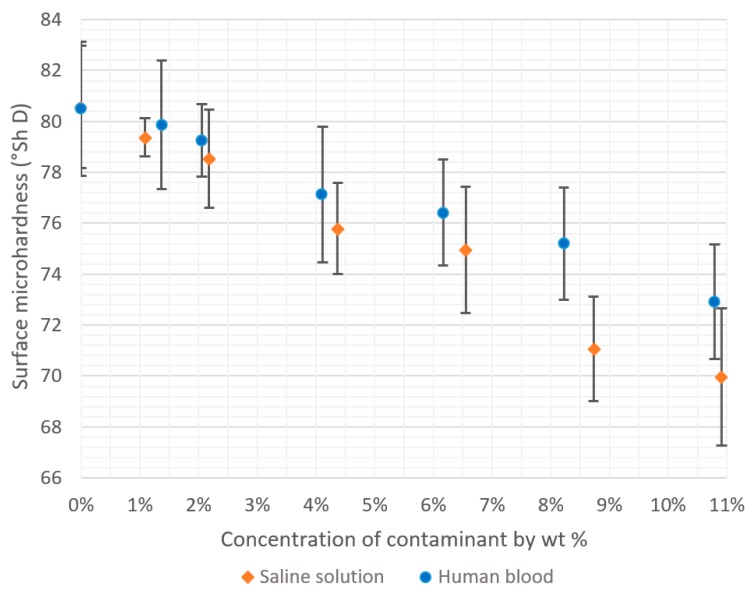
Decrease in hardness of bone cements relative to the degree of contamination with physiological fluids.

The results of the hardness tests of the cement specimens of varying degrees of physiological fluid contamination are presented in Table 3 and Table 4. Similarly to the compressive strength analysis, all measurements show a negligible scatter of results and the R^2^ factor is contained below 4%, which indicates a high homogeneity of the examined indicator. Similarly, in this case, the decrease in the average hardness of the cement samples is evidently greater for the saline solution compared with the human blood. A graphical summary of the results is given in Figure 5, whereas the detailed statistical analyses of the obtained results are presented in the Tables below.

### 3.3. Statistical Analysis

Table 5 and Table 6 present the results from the statistical analysis, which set out to verify the equality of mean cement compressive strength relative to the amount of contamination in the tested substrates, in accordance with the adopted level of statistical significance α = 0.05.

In general, the observations confirm the data from the preliminary graphs of the mean strength values. The impact of blood contaminants present in the bone cement material is reflected in the severe decrease in its compressive strength throughout the analysed range; however, it is the most radical at the lowest dosages. For progressive cement contamination with human blood, a statistically relevant reduction in strength was observed up to the level of approx. 2 wt% of blood in cement (uncontaminated vs. 1.4%/2.1%, *p* = 0.00). In higher concentrations, the mean strength values recorded in the tests oscillated around 70 MPa, i.e., the value specified in ISO 5833: 2002 as the minimum required compressive strength of cement (for example, 2.1% vs. 4.1%, *p* = 0.50 and 4.1% vs. 6.1%, *p* = 0.53, etc.; see Table 5). From this, it can be concluded that blood, ever-present throughout the prosthesis implantation procedure, does not have a significant negative impact on its basic strength characteristic—the mechanical strength of the cement material—i.e., it does cause a statistically significant decrease in strength; however, only in certain measurements of highly-contaminated samples, and the readings are below the indicated range of approx. 69–70 MPa.

Nevertheless, the presence of physiological saline solution, also unavoidable during surgery, e.g., as a coolant, is notably different. From the statistical analysis, it becomes clear that the statistically significant differences were almost always recorded between the individual values of mean compressive strength recorded in tests. Thus, this is a solid confirmation that the strength of the cement material is highly susceptible to the presence of a saline solution, which shows a significant and progressive decrease as the amount of the solution increases. Cement degradation is greater at the initial dosages, i.e., in the range of 1% to 5% saline solution contamination (almost always *p* = 0.00—see Table 6). Subsequently, it decelerates and becomes more stable in the range of 8% to 10%, where the decrease in the compressive strength of the cement specimens was rather constant (*p* = 0.17). An important observation to note is that in specific conditions, with the inclusion of 5–6 wt % of saline solution, the strength of cement specimens falls below the 70 MPa limit specified in the ISO standard. Secondly, regardless of the type of contamination, at the top boundary of the contaminant level (8%–10%), the cement’s compressive strength falls below the said acceptable limit.

The results from the tests verifying the equality of means of cement hardness are presented in the following Table 7 and Table 8.

From the above comparison, it can be seen that with respect to human blood, a decrease in response to lower-degree contamination does not affect the statistical significance of the change in cement hardness (for example, uncontaminated vs. 1.4%, *p* = 0.58, and vs. 2.1%, *p* = 0.21, etc.; see Table 7). Moreover, it remains virtually unchanged throughout the entire tested range (*p* > 0.05—see Table 7). Although the disparity is recorded, it does not concern the neighbouring values. The explanation for this distribution of results should be sought not so much in the inaccuracy of measurements, as it was shown to be small within the particular series (CV: blood 1.8%–3.5%, salt 0.9%–3.8%), but rather in high resistance of the cement material to blood contamination. Regarding compressive strength, the hardness of cement contaminated with saline shows a greater affinity to the concentration of contamination than in the case with human blood. The statistically significant differences were already shown even between successive measuring points (2.2% vs. 4.4%, *p* = 0.01 or 6.6% vs. 8.7%, *p* = 0.01). A small increase in the amount of the physiological saline solution leads to a considerable decrease in hardness. This is, moreover, confirmed by the models reported later in this work.

### 3.4. Mathematical Modelling

The quantitative comparison of the effect that the two analysed factors exert on the bone cement degradation was performed with the application of mathematical modelling. The first analysed relationship was the dependence of mean hardness on the amount and type of contamination, as the results from the experiments indicated that it could be described by simple linear models (Equation (1), Table 9).

*hardness* = *m* × x + b, (1)

where x is the amount of contaminant by wt %.

From the comparison, it can be clearly seen that the obtained models exhibit a good fit with the experimental data (coefficient of determination R^2^ = 0.983/0.987). Considering the slope coefficients (m), it was shown that the analysed cement is approx. 30% more sensitive to the change in the concentration of physiological saline by mass than to the increased blood contamination.

Nevertheless, the compressive strength of the contaminated cement could not be represented with the linear model, as its incapacity for that purpose was particularly evident in the case of the test specimen series contaminated with human blood (R^2^ = 0.59—a weak fit). Other typical mathematical models (exponential, logarithmic or power) were also proved unsuitable. In the case of a polynomial model, implementing additional polynomial powers into the model considerably improved the fit (Equation (2), Table 10). For the third-degree polynomial model, the final coefficients of determination were R^2^ = 0.88 (blood) and R^2^ = 0.92 (saline solution). A further expansion of the polynomial model might produce even more accurate matching, however, due to the inherent instability of these models, i.e., fluctuations in the fitness of values, which although show very good fit in the range, are nonetheless susceptible to rapid deterioration outside the considered data range (typical problems with models for precise prediction of the further course of the function).

*Compression strength* = α_1_ × x^3^ + α_2_ × x^2^ + α_3_ × x + ε, (2)

where x is the amount of contaminant by wt %.

**Figure 6:**
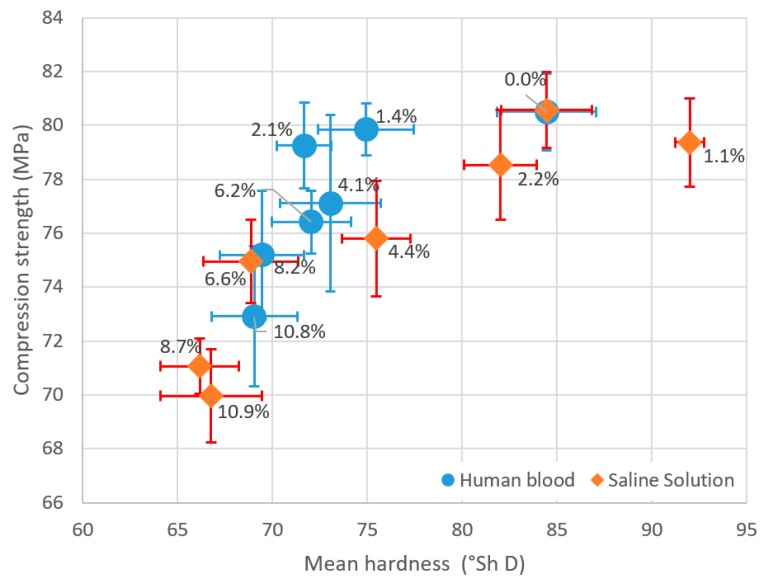
Summary of the strength characteristics degradation in contaminated cement.

Based on existing models [40,41,42,43] that combine the mechanical properties of selected materials, the evaluation of the analysed mechanical properties of the tested cement was attempted. The results from this analysis, given in Figure 6, clearly show the course of simultaneous degradation of both strength characteristics of the test material. Despite persistent attempts to match a fitting mathematical relationship to simultaneously account for the compressive strength and hardness of cement, no satisfactory results were obtained. Hence, it was resolved that they would not be presented in the paper.

## 4. Discussion

Bone-cement contamination is a problem whose effects have not yet been thoroughly studied or presented in the specialist literature. In the study by Tan et al. [34], it was shown that the rate of strength characteristics degradation of blood-contaminated cements was significantly greater when the composition of the cement contained gentamicin than with its absence. The results from their study are particularly interesting given that bone-cement compositions with antibiotics introduced by the manufacturer are widely used in Europe, whereas in the United States manual antibiotic powder addition to traditional bone cement during surgery is preferred [44]. The authors of other studies [34,45] monitored the formation of gaps and voids in the micromorphological structure of the blood-contaminated cement, whose primary effect is the reduction in the cement shear strength. On the other hand, the test results presented in the study by Graham et al. [46] indicated the positive effect of the suitable selection of the cement-mixing method (vacuum vs. manual) on the reduction of porosity, which were found to increase the crack resistance and its cyclic loading strength. Centrifugation and vacuum mixing were under investigation in the work of Sayeed et al. [32], which found that the methods in question significantly reduce the introduction of air into the mix and thus curb the formation of porosity in the cement structure. As a result, the compressive strength and energy absorption capacity of cement materials are increased. The application of compressive loading on the cement paste upon filling the femur was found to bring equally positive results in several studies [47,48]. Bone cement is a fragile material whose strength is highly susceptible to internal stresses emerging as a consequence of cavity formation in the material structure [49]. Voids in the cement structure, which are either empty or filled with clotted blood, saline solution or salt, do not transfer stress in the material and, as a result, cause degradation of cement strength properties. The extent of the degradation depends on the quantitative degree of contamination (by mass). However, a similar extent of contamination with various factors has been shown to degrade both the compressive strength and cement hardness to varying degrees. Nevertheless, considering the limit of contamination (blood 7%, saline 5.5%), the strength eventually falls below the minimum strength of 70 MPa—required of the cement material as specified in the International Standard ISO 5833: 2002. In two studies of 1980s cement material (IMPLAST) [49,50], 20% of the monomer was replaced with water, which produced a highly porous structure and resulted in significant degradation of the material’s resistance to cyclic (fatigue) loading.

Although the stress–strain curves in Figure 4 represent only selected individual specimens—representatives of each series—it should be noted that the cement behaviour under loading is strongly contamination-dependent, which is also observed for the remaining specimens in a batch. The slope of the curve in the elastic deformation zone is noticeably greater in the case of contamination with saline compared to blood. However, the change in the concentration of blood does not lead to such a marked change in the slope as in the case with physiological saline.

The importance of compressive strength tests follows from the fact that cement is subjected to loading during normal operation of the prosthesis not only by significant static forces, resulting from maintaining the weight of the patient standing, but also from dynamic forces occurring during movement, particularly given that the latter can be quite significant. The maximum reactions, determined by means of measuring implants in a regular walk, are from 250% of body weight (BW) for the hip joint (HJ), and up to 260% of body weight for the knee joint (KJ). When descending stairs, it is, respectively, 260% BW for HJ and as much as 350% BW for KJ. When squatting, the values of reaction forces are, respectively, 150% BW for HJ and 250% BW for KJ [51,52]. Stresses occur in the cement range from 3–11 MPa, relative to the cement thickness and the performed activity [15,53].

Hardness, in turn, combines several material properties: The resistance to deformation, friction, abrasive wear or fracture propagation. Mathematical models have been developed that allow converting hardness parameters to determine, e.g., the cement tensile strength [43]. Although it should seem that in bone cements the problem of friction is non-applicable, in the event of backlash in the bone-cement-implant system, friction processes may occur to a certain extent. In such scenarios, the abrasion resistance of the cement, directly related to the hardness of the material, will become its most critical feature. The wear of cement damaged in the given circumstances can be defined as the loss of material, causing cement particles to penetrate neighbouring soft tissues. The important derivative processes to account for would be the peripheral deformation and further degradation. Alternatively, when as a result of regular operation cement begins to crack, its strength (compressive strength or hardness) will depend on whether and how the crack propagates.

The numerical models employed in the study established the mathematical relationships between the specific cement contamination concentrations and the qualitative degradation of its strength properties. Furthermore, given their good fit with the experimental data, they should be considered as accurate. The R^2^ coefficient of determination for the linear hardness models did not exceed 0.98 and for the polynomial (third-degree) models of compressive strength ranged from 0.88 to 0.92. In the available specialist literature in the field, mathematical modelling is rarely used, which results from either the narrow scope of studies or a small variability of the analysed parameters.

Although there are certain similarities between our study and, e.g., Wiegand et al. [15], the latter concerns the intentional chemical modification of the cement by supplying inorganic material, usually in the form of particles, fibres or bioactive minerals. In another study [1], cements were supplemented with synthetic hydroxyapatite (HA) (i.e., a mineral that is found, e.g., as a component of bones and teeth [54,55,56], and is implemented as a bone growth stimulator in small bone defects and as a coat for implants (e.g., hip endoprosthesis)). No statistically significant differences were observed between contaminated and uncontaminated cements, even given the extensive range of contaminant concentration, 20%–40% by volume. This unexpected resistance to the contaminant admixture results from the uniform distribution of HA particles in the cross-linked PMMA structure, which simultaneously leads to a significant increase in the value of the compressive modulus. Additives in the form of carbon nanotubes have also been investigated as a solution to the presence of contamination in the operated area: These structures form bridges and thus prevent crack propagation increasing the fracture toughness [13]. In a different study, it was revealed that the incorporation of silica nanotubes increased the bending modulus and the compressive strength with increasing concentrations, whereas the flexural strength and the fracture toughness decreased [15,35]. The hydrolysis-resistant titanium–bone cement interface, has been, furthermore, proven to counteract aseptic loosening by modifying the cement substrate with methacryloxypropyl-trimethoxysilane [48]. Other bone cement additives that have been put to test are microhydroxyapatite, -magnesium oxide, -barium sulphate and -silica particles [15,36,37], as well as methacrylate crosslinkers ethylene glycol-dimethacrylate (EG-DMA) [15,57,58] or hydroxyethyl methacrylate (HEMA) [15,59], and finally, triethylene glycol-dimethacrylate (TEG-DMA) [15,60,61]. PMMA cement supplementation with vancomycin was assessed in the study by Ajit Singh et al. [30], who concluded that even a 2 g addition of the drug per a 40 g container of cement (5% by weight) significantly affects the degradation of the three-point bending flexural strength. The antibiotic, supplemented in the form of a powder acts as an inclusion that causes stress build-up and as a result the weakening of the cement [30,62]. The study also tested the antibacterial properties of the antibiotic-supplemented cement: The increase in the amount of antibiotic in the mix, except for not contributing to any improvement in the bactericidal effectiveness, furthermore accelerated its mechanical degradation.

The thinning of the cement mass with liquid [49] or intentional feeding of solid contaminants [38] will also modify and reduce the temperatures generated during the exothermal polymer crosslinking.

As specified in the plan of the study, the tests analysed the effect of the presence of the indicated contaminants on the degradation of the bone cement static strength parameters. Further research should continue and extend the scope of analyses reported in this paper, in particular, with respect to simultaneous contamination with blood and saline solution—the actual conditions during surgery are never invariable, and contamination does not occur separately. Secondly, the future approach ought to account for the variable cyclic loading strength, which was beyond the scope of this or former investigations, and which reflects the actual conditions of the cement prosthesis operation [63]. With a view to reducing the number of tests and simplifying the study, additional parameters that have a bearing on the cement strength and the inclusion of which would more closely reflect the actual conditions of the cement operation were also omitted. This includes the problem of cement aging over time, which could be incorporated in the future research plans [64,65], along with other factors such as the impact of the human body conditions on the strength characteristics of prosthetic materials, which could be simulated by means of seasoning in Ringer’s solution [22] or saline solution [66]. Finally, it must be highlighted that in the reported analyses, the cement samples were subject to testing at an ambient temperature lower than the average regular human body temperature, which determines a further area of experimental exploration in the field.

## 5. Conclusions

This study showed a statistically significant effect of physiological fluid contamination on the deterioration of the mechanical properties of bone cements. The cement specimens were shown to achieve sub-critical strength (below the 70 MPa recommended in the International Standard) when contaminated with the selected physiological fluids. The conditions in question were observed in samples where blood constituted more than 7 wt % of the cement mass, whereas in the saline-contaminated material the threshold was approx. 5.5 wt %. The cement hardness was shown to degrade linearly and, what is more, the comparative analysis of the two tested contaminants proved that it was the physiological saline solution that caused the higher degree of cement material hardness degradation, amounting to approx. 30%. It should appear that the data regarding the effect of contamination with blood and other bodily fluids on the strength of the cement and cement bond would be readily available; however, the manufacturer fails to provide specific data, particularly regarding the quantitative composition of the cement mix. The sole communication on the part of the manufacturer concerns a warning for the surgeon included in the material specifications and application recommendations. It is suggested that the bone cavity should be thoroughly cleaned prior to the deposition of the bone cement, including brushing and rinsing for the removal of adipose tissues, bone marrow and other contaminants. The cavity is to remain clean in order for blood and other contaminants not to become mixed with the cement substrate. Nevertheless, due to the conditions in the operating room, particularly in hospitals, and economic factors, some orthopaedists are unable to adhere to these recommendations. Our study shows that the 3%–5% contamination by volume significantly modifies the cement properties. It should be noted that the cement layer that is in direct contact with both physiological fluids (blood, synovial fluid and fat) and 0.9% NaCl solution (used to rinse the joint before prosthesis implantation with cement) could be contaminated to a much further extent than the range considered in this study. Given the paucity of reliable information from the manufacturer that would accurately describe these effects, our study set out to approach this problem, which is expected to carry more far-reaching implications of cement contamination than realised by orthopaedists, i.e., its effect on the proper strength characteristics, its reliability in the longer time perspective and the problem of aseptic loosening of implants and all other related consequences.

## Figures and Tables

**Table 1:** Compressive strength of cement specimens relative to the degree of blood contamination.

Contamination Level	Mean Compression Strength (MPa)	SD (MPa)	CV
Human blood			
0.0%	84.45	1.42	1.7%
1.4%	74.94	0.97	1.3%
2.1%	71.68	1.59	2.2%
4.1%	73.06	3.28	4.5%
6.2%	72.04	1.16	1.6%
8.2%	69.45	2.40	3.5%
10.8%	69.05	2.60	3.8%

**Table 2:** Compressive strength of cement specimens relative to the degree of physiological saline solution contamination.

Contamination Level	Mean Compression Strength (MPa)	SD (MPa)	CV
Saline solution			
0.0%	84.45	1.42	1.7%
1.1%	92.00	1.63	1.8%
2.2%	82.02	2.04	2.5%
4.4%	75.48	2.14	2.8%
6.6%	68.86	1.55	2.3%
8.7%	66.17	1.03	1.6%
10.9%	66.77	1.72	2.6%

**Table 3:** Hardness of cement specimens relative to the degree of blood contamination.

Contamination Level	Mean Hardness	SD (°Sh D)	CV
	(°Sh D)		
Human blood			
0.0%	80.50	2.63	3.3%
1.4%	79.86	2.53	3.2%
2.1%	79.25	1.43	1.8%
4.1%	77.11	2.66	3.5%
6.2%	76.42	2.09	2.7%
8.2%	75.19	2.21	2.9%
10.8%	72.91	2.25	3.1%

**Table 4:** Hardness of cement specimens relative to the degree of saline contamination.

Contamination Level	Mean Hardness	SD (°Sh D)	CV
	(°Sh D)		
Saline solution			
0.0%	80.57	2.40	3.0%
1.1%	79.37	0.75	0.9%
2.2%	78.53	1.92	2.4%
4.4%	75.80	1.80	2.4%
6.6%	74.95	2.49	3.3%
8.7%	71.07	2.05	2.9%
10.9%	69.97	2.68	3.8%

**Table 5:**
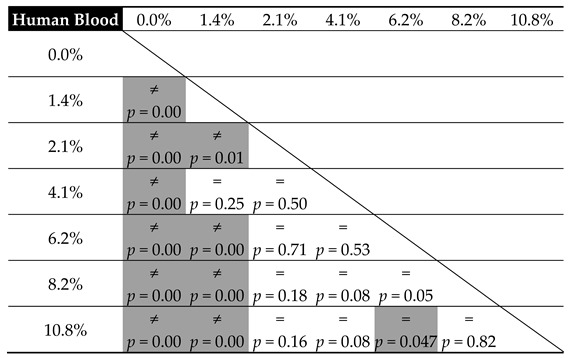
Equality of mean cement compressive strength relative to the degree of blood contamination.

**Table 6:**
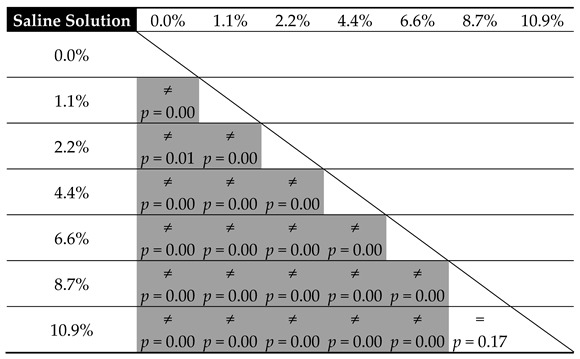
Equality of mean cement compressive strength relative to the degree of physiological saline solution contamination.

**Table 7:**
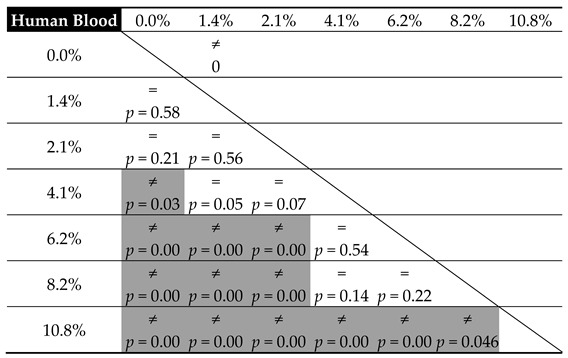
Equality of means of cement hardness relative to blood contamination.

**Table 8:**
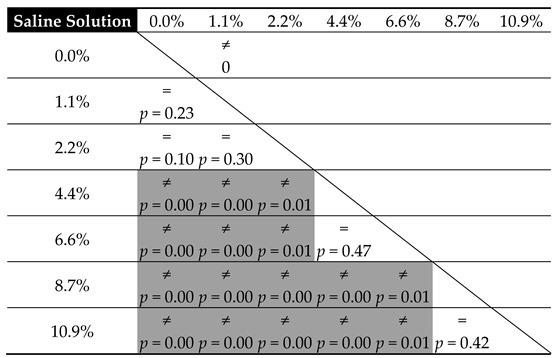
Equality of means of cement hardness relative to physiological saline solution contamination.

**Table 9:** Hardness of the contaminated cement—the linear model.

Contaminant	Parameters of the Linear Model of Hardness Change (m × x + b)		
	m	b	R^2^
Human blood	−69.48	80.57	0.987
Saline solution	−99.32	80.55	0.983

**Table 10:** Compressive strength of the contaminated cement—the polynomial model.

Contaminant	Parameters of the Polynomial Model of Compression Strength Change (α_1_ × x^3^ + α_2_ × x^2^ + α_3_ × x + ε)					
	Polynomial model degree	α_1_	α_2_	α_3_	ε	R^2^
Human blood	1st (linear)	-	-	−102.76	78.33	0.59
	2nd (quadratic)	-	1716	−285.58	80.87	0.75
	3rd (cubic)	−56,651	10,954	−659.22	83.20	0.88
Saline solution	1st (linear)	-	-	−223.85	87.35	0.84
	2nd (quadratic)	-	1540	−388.87	89.54	0.87
	3rd (cubic)	61,137	−8523	21.18	87.12	0.92
